# Toward the development of a molecular toolkit for the microbial remediation of per-and polyfluoroalkyl substances

**DOI:** 10.1128/aem.00157-24

**Published:** 2024-03-13

**Authors:** Miao Hu, Colin Scott

**Affiliations:** 1 CSIRO Environment, Black Mountain Science and Innovation Park, Canberra, ACT, Australia; Danmarks Tekniske Universitet, The Novo Nordisk Foundation Center for Biosustainability, Kgs. Lyngby, Denmark

**Keywords:** PFAS, defluorination, microbial remediation, defluorinase, dehalogenase

## Abstract

Per- and polyfluoroalkyl substances (PFAS) are highly fluorinated synthetic organic compounds that have been used extensively in various industries owing to their unique properties. The PFAS family encompasses diverse classes, with only a fraction being commercially relevant. These substances are found in the environment, including in water sources, soil, and wildlife, leading to human exposure and fueling concerns about potential human health impacts. Although PFAS degradation is challenging, biodegradation offers a promising, eco-friendly solution. Biodegradation has been effective for a variety of organic contaminants but is yet to be successful for PFAS due to a paucity of identified microbial species capable of transforming these compounds. Recent studies have investigated PFAS biotransformation and fluoride release; however, the number of specific microorganisms and enzymes with demonstrable activity with PFAS remains limited. This review discusses enzymes that could be used in PFAS metabolism, including haloacid dehalogenases, reductive dehalogenases, cytochromes P450, alkane and butane monooxygenases, peroxidases, laccases, desulfonases, and the mechanisms of microbial resistance to intracellular fluoride. Finally, we emphasize the potential of enzyme and microbial engineering to advance PFAS degradation strategies and provide insights for future research in this field.

## INTRODUCTION

Per- and polyfluoroalkyl substances (PFAS) are anthropogenic, aliphatic organic substances with at least one fully (per-) or partly (poly-) fluorinated carbon chain (1). PFAS have been widely employed in numerous industrial and consumer applications since the 1950s due to their exceptional physicochemical properties, including hydrophobicity, lipophobicity, and thermal stability (2–4). The proliferation of PFAS and their diverse applications has exhibited significant growth over time.

**Fig 1:**
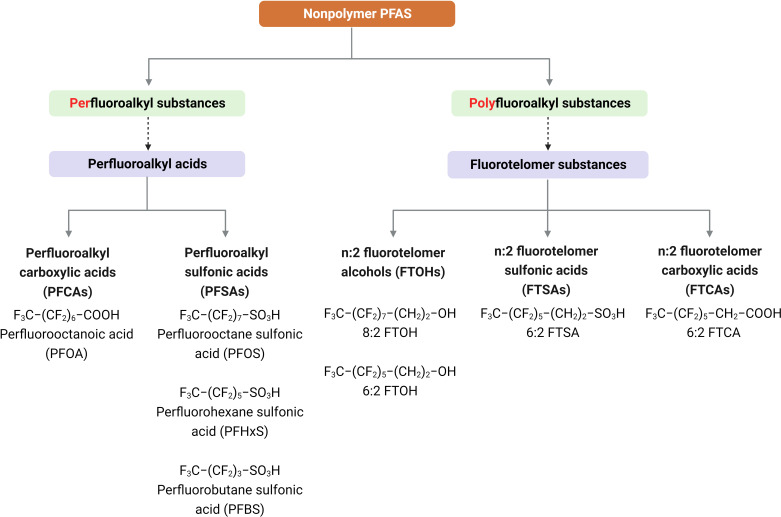
Classification of PFAS compounds. Abbreviations and structures of PFAS subclasses are mentioned in this review. This figure was created with BioRender.com.

The PFAS family represents a broad and diverse chemical family. However, of the 4,730 PFAS with CAS registry numbers, only 256 are considered commercially relevant (5, 6). Buck et al. (2) divided PFAS into two primary classes: non-polymers and polymers. Within the non-polymer PFAS class, two major subclasses have been identified: perfluoroalkyl substances and polyfluoroalkyl substances, which comprise numerous groups and subgroups of chemicals. The perfluoroalkyl substances, specifically perfluoroalkyl acids, can be further divided into two major subgroups: perfluoroalkyl carboxylic acids (PFCAs) [e.g., perfluorooctanoic acid (PFOA)] and perfluoroalkyl sulfonic acids (PFSAs) [e.g., perfluorooctane sulfonic acid (PFOS), perfluorobutane sulfonic acid (PFBS), and perfluorohexane sulfonic acid (PFHxS)]. Additionally, within the polyfluoroalkyl substances, the fluorotelomer substances include subgroups: n:2 fluorotelomer alcohols (FTOHs) (e.g., 8:2 FTOH and 6:2 FTOH), n:2 fluorotelomer sulfonic acids (FTSAs) (e.g., 6:2 FTSA), and n:2 fluorotelomer carboxylic acids (FTCAs) (e.g., 6:2 FTCA) (Fig. 1).

Due to the extensive use and consequent discharge, PFAS have been identified in a range of environmental contexts, such as drinking water (7, 8), surface and groundwater (9, 10), soils (11, 12), wastewater (13, 14), landfills (15), remote areas (far from any local point sources, e.g., Arctic environment) (16, 17), and agricultural plants (18). Consequently, both wildlife and humans are subject to exposure to PFAS contamination (19–24). Numerous epidemiological and mechanistic investigations have implied a connection between exposure to PFAS and a wide range of diseases, such as altered immune function, adverse reproductive effects, liver and kidney damage, obesity, and cancers (25–31).

The numerous physical and chemical treatment options for PFAS that have been developed are often accompanied by limitations, including high costs, high energy use, generation of hazardous transformation byproducts, and the need for additional post-processing (32). In contrast, biodegradation provides a potentially favorable alternative approach for achieving complete PFAS degradation via a low-cost, environmentally friendly technology. Biodegradation using microbes or cell-free enzymes has been demonstrated to be technically effective for a range of contaminants, including herbicides (33), insecticides (34, 35), fungicides (36–38), and industrial solvents (39, 40). The biodegradation of naturally occurring and synthetic organochlorides has been investigated and documented extensively (41, 42). However, for organofluorides, particularly PFAS, biodegradation has been rarely demonstrated in natural environments.

The paucity of PFAS defluorination in nature can be attributed to the following factors. First, the carbon-fluoride (C-F) bond is exceptionally strong compared to other carbon-halide bonds (43). Second, polyfluorinated products are not known in nature, and only a few naturally occurring monofluorinated compounds have been observed (44, 45). This suggests that until the recent introduction of anthropogenic organofluorides, there was no strong selection pressure for the catabolism of these compounds, the exception being for highly toxic natural organofluorides such as fluoroacetate. Third, sustained biodegradation of PFAS is likely dependent on the presence of multiple systems, including low-potential redox transfer proteins, defluorinases, transport mechanisms for substrate uptake, fluoride export proteins, and enzymes with inherent resistance to fluoride inhibition (46, 47). Recently, numerous studies have been conducted and reported on the biotransformation and biodegradation of PFAS, focusing on the release of fluoride and the identification of reaction products. However, the identification of specific microorganisms or enzymes that catalyze the defluorination of PFAS remains limited (1, 48–50).

Here, we provide a thorough overview of the enzymes that have been suggested to be responsible for the catabolism of PFAS thus far. The enzymes under consideration include haloacid dehalogenases, reductive dehalogenases, cytochromes P450, alkane and butane monooxygenases, peroxidases, laccases, and desulfonases. The PFAS-related reactions catalyzed by these enzymes are summarized in Table 1. Drawing from the enzymes displaying activity against PFAS, a perspective is presented on the advancement of enzyme and microbial engineering strategies for the catabolism of PFAS, offering insights into potential future directions for research in this field.

**TABLE 1:** Enzyme-induced reactions for biotransformation of PFAS (med and med^−^ are the oxidized and reduced forms of small molecule mediators used in peroxidase- and laccase-mediated reactions)

Enzymes	Reactions
Haloacid dehalogenases	**F**-CH_2_-COOH + H_2_O → **OH**-R-CH_2_-COOH + H^+^ + F^−^
Reductive dehalogenases^*a*^	**F**-R-CH_2_-COOH + 2e^−^ + H^+^ → **H**-R-CH_2_-COOH + F^−^
Cytochromes P450, monooxygenases^*a*^	**F**-C-R + O_2_ + 2e^−^ + 2H^+^ + NAD(P)H → **OH**-C-R + NAD(P)^+^ + H_2_O + H^+^ + F^−^
Peroxidases^*a*^	**2** F-C-R + med + H_2_O_2_ → **F**-C-R + 2med^−^ + 2H_2_O → 2med-C-R + 2F^−^ +2H_2_O
Laccases^*a*^	**4** F-C-R + 4e^−^ + 4med + O_2_ → **F**-C-R + 4med^−^ + 2H_2_O → 4med-C-R + 4F^−^ + 2H_2_O
Desulfonases^*a*^	**F**-R-CH_2_-SO_3_H + FMN + NAD(P)H + H^+^ → **F**-R-CHO + FMN + NAD(P)^+^ + H^+^ +SO_3_^2−^

^*a*^ Reactions mediated by these enzymes are proposed only; the strength of the evidence for each is described in the text.

## ENZYMES INVOLVED IN PFAS DEFLUORINATION

### Haloacid dehalogenases

Haloacid dehalogenases are a class of hydrolases that specialize in hydrolytic cleavage of carbon-halide bonds (51). Bacterial 2-haloacid dehalogenases can be categorized into three groups according to their substrate specificities: L-2-haloacid dehalogenase, D-2-haloacid dehalogenase, and DL-2-haloacid dehalogenase. The L-2-haloacid dehalogenases exhibit the ability to catalyze the hydrolytic dehalogenation of L-2-haloalkanoic acids, resulting in the formation of the corresponding D-2-hydroxyalkanoic acid. Since the 1980s, L-2-haloacid dehalogenases have been identified, isolated, and extensively characterized in detail (52, 53).

2-Fluoroacid dehalogenases (often referred to as fluoroacetate dehalogenases) exhibit enzymatic defluorinase activity and often possess activity against other haloalkanoic acids, such as chloroacetate, albeit often with lower efficacy than with fluoroacetate (54–56). Throughout this review, the term “fluoroacetate dehalogenases” was consistently employed for haloacid dehalogenases annotated as such to remain consistent with published literature and database entries.

Fluoroacetate is a natural organofluoride compound that is toxic to mammals and produced by actinomycetes, such as *Streptomyces cattleya* (57), and acts as a defense molecule in some plants (58, 59). Fluoroacetate dehalogenase catalyzes the hydrolytic cleavage of carbon fluoride in fluoroacetate, yielding glycolate and fluoride. These enzymes have been isolated in phylogenetically diverse bacteria, such as *Pseudomonas* (60–63), *Delftia acidovorans* (formerly *Moraxella*) (64, 65), *Burkholderia* (56), and *Rhodopseudomonas* (66). Some fluoroacetate dehalogenases, e.g., Fac-DEX H-1 (H-1), Fac-DEX FA1 (FA1), and RPA1163, have undergone comprehensive characterization. Their basic properties are listed in Table 2. The FA1 shared a sequence identity of 61% with H-1 (56) and 42% with RPA1163 (67). Furthermore, the crystallographic findings reveal that both FA1 and RPA1163 are homodimer proteins, belonging to α/β hydrolase superfamily (67, 68).

**TABLE 2:** Representative haloacid dehalogenases responsible for defluorination^*a*^

Protein name	RPA1163	FAc-DEX	H-1	POL0530	RJO0230	DAR3835	NOS0089
Protein length	302 aa	304 aa	294 aa	229 aa	254 aa	303 aa	291 aa
Gene name	RPA1163	fac-dex	dehH1	Bpro0530	RHA1_ro00230	Daro3835	Alr0039
Uniprot ID	Q6NAM1	Q1JU72	Q01398	Q12G50	Q0SK70	Q479B8	Q8Z0Q1
PDB code	3R3U	1Y37	Not reported	3UM9	3UMG	8SDC	3QYJ
Microorganisms	*Rhodopseudomonas palustris* CGA009	*Burkholderia* sp. FA1	*Delftia acidovorans* B (formerly *Moraxella* sp. B)	*Polaromonas* sp.	*Rhodococcus jostii* strain RHA1	*Dechloromonas aromatica*	*Nostoc* sp.
Substrates	Fluoroacetate, difluoroacetate, chloroacetate, 2-fluoropropionic acid, 2,3,3,3-tetrafluoropropionic acids	Fluoroacetate, chloroacetate, bromoacetate	Fluoroacetate, chloroacetate, bromoacetate, iodoacetate	Fluoroacetate	Fluoroacetate, 6:2 FTOH, 6:2 FTSA	Fluoroacetate, difluoroacetate, 2,2-difluoropropionic acid, 5,5,5-trifluoropentanoic acid	Fluoroacetate, difluoroacetate, 2,2-difluoropropionic acid, 5,5,5-trifluoropentanoic acid
References	(66, 67, 69, 70)	(56, 68)	(55, 64, 65, 71)	(66, 72)	(66, 72, 73)	(66, 74)	(66, 74)

^*a*^ aa, amino acids.

There are also haloacid dehalogenases that possess the capacity to catalyze the hydrolytic dehalogenation reactions on a range of haloacetates that do not include fluoroacetate. For example, haloacid dehalogenase H-2 (H-2) (Uniprot ID: Q01399), isolated and purified from *D. acidovorans* B (formerly *Moraxella* sp. B), can dehalogenate chloroacetate, bromoacetate, and iodoacetate, and has some catalytic activity on 2,2-dichloroacetate and 2-chloropropionate (54, 65). Despite being present on the plasmid pUO1 in *D. acidovorans* B, the genes *dehH1* and *dehH2*, which, respectively, encode enzymes H-1 and H-2, exhibit no sequence homology (75, 76). This lack of homology suggests that enzymes H-1 and H-2 do not share a common evolutionary origin (76).

Some novel L-2-haloacid dehalogenases with defluorination activity were found through sequence- and activity-based screening of microbial genomes, including POL0530 (from *Polaromonas* sp. strain JS666) and RJO0230 (from *Rhodococcus jostii* strain RHA1) (strain RHA1), DAR3835 (from *Dechloromonas aromatica*), NOS0089 (from *Nostoc* sp.), and POL4478 (from *Polaromonas* sp.) (66) (Table 2).

**Fig 2:**
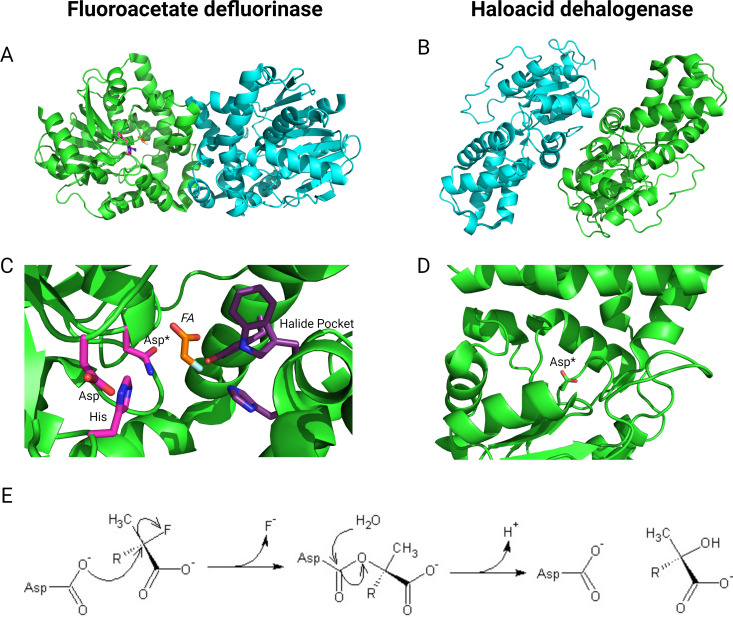
Hydrolytic defluorinases. Cartoon representation of the dimeric structures of the fluoroacetate dehalogenase (**A**) and defluorinating haloacid dehalogenase (**B**) from *Rhodopseudomonas palustris* (PDB 3R3W) (67) and *Polaromonas* sp. JS666 (PDB 3UM9) (PDB DOI: https://doi.org/10.2210/pdb3um9/pdb), respectively. The homodimers are shown with the two identical chains shown in green and cyan. A more detailed view of the active sites is shown (**C and D**) with the nucleophilic aspartate residue shown (Asp*, this is an Asn residue in the Asp110Asn variant of fluoroacetate dehalogenase shown). The substrate is bound in fluoroacetate dehalogenase (*FA*, orange), and the residues that stabilize the charge developed on the fluoride (halide pocket, purple) are also shown. The reaction mechanism is shown (E; an S_N_^2^ nucleophilic attack, followed by the formation of a tetrahedral intermediate and regeneration of the nucleophile via attack by water). This figure was created with BioRender.com.

The catalytic mechanism for hydrolysis of fluoroacetate by haloacid dehalogenases is an S_N_^2^ displacement reaction involving two steps, namely, the formation of an enzyme-ester intermediate followed by the subsequent hydrolysis of the ester intermediate via an activated water molecule (71, 77) (Fig. 2). A catalytic mechanism was suggested based on studies conducted on the fluoroacetate dehalogenase H-1 (71). Site-directed mutagenesis and molecular simulations demonstrated the essential role of aspartate (Asp-105; H-1 numbering) and histidine (His-272; H-1 numbering) in the catalytic process. Asp-105 serves as a nucleophile to attack the α-carbon of fluoroacetate, leading to the release of a fluoride anion and the formation of an enzyme-ester intermediate. The latter was subsequently hydrolyzed by a water molecule activated by His-272 (71).

The suggested mechanism was supported by structural, quantum mechanical/molecular mechanical (QM/MM), and site-directed mutagenesis studies with the fluoroacetate dehalogenase FA1 (68, 78, 79). The catalytic mechanism of FA1 depends on a catalytic triad, Asp104-His271-Asp128 (FA1 numbering), where Asp104 acts as a nucleophile, facilitating the removal of the fluoride anion from fluoroacetate (68). Meanwhile, QM/MM calculations indicated that Asp128 stabilizes the transition state for the transfer of a proton from a water molecule to His271 and the subsequently formed tetrahedral intermediate (78). Additionally, the use of QM/MM calculations provides insights into the potential contributions of various amino acids within the active site of FA1. Water molecules and the surrounding amino acid residues (Arg105, Arg108, His149, Trp150, and Tyr212) form a hydrogen-bonding network that positions fluoroacetate for hydrolysis. His149, Trp150, and Tyr212 play a significant role in stabilizing the leaving fluoride atom (78, 79). In particular, Trp150 plays a crucial role specifically in the defluorination of fluoroacetate, while its involvement is not necessary for the dechlorination of chloroacetate. This distinction highlights the importance of a hydrogen-bond interaction between Trp150 and the fluorine atom of fluoroacetate, as it is shown to be an absolute requirement for effectively reducing the activation energy associated with the cleavage of the strong C-F bond (68, 78).

Similarly, the crystal structure of fluoroacetate dehalogenase RPA1163 revealed the presence of a catalytic triad (Asp110-His280-Asp134; RPA1163 numbering) and an active site composition that is identical to that of FA1 and confirming the functional roles of His155, Trp156, and Tyr219 residues in stabilization of the leaving fluoride (67). Tyr219 functions as a charge acceptor along the S_N_2 reaction axis, thereby mitigating electronic repulsion during the nuclophilic substition (80). Yue et al. (70) conducted QM/MM calculations to propose a comprehensive mechanism for the catalytic degradation of fluoroacetate by RPA1163, followed the same mechanism as the other fluoroacetate defluorinases, and identified that the rate-determining step for fluoroacetate degradation is the nucleophilic attack.

The recent determination of the crystal structure of haloacid dehalogenases POL0530 and RJO0230 has unveiled a remarkable similarity in their overall structure and active site composition (Fig. 2). Furthermore, molecular dynamics calculations indicate that these defluorinating enzymes adopt more compact conformations than their non-defluorinating counterparts, facilitating enhanced interactions with the fluorine atom and thus increasing their efficiency (72).

Multiple theoretical investigations have suggested that it may be possible to use RPA1163 for the defluorination of fluorinated compounds in addition to the presumed physiological substrate, fluoroacetate. To validate the theoretical outcomes, *in vivo* experiments were subsequently conducted, providing empirical evidence for the substrate promiscuity in RPA1163. For example, Wang et al. (81) demonstrated that RPA1163 effectively defluorinates bulky substrates such as 2-fluoro-2-phenyl acetic acid and 2-fluoro-2-benzyl acetic acid. In addition to 2-fluoropropionic acid, Li et al. (69) also observed the degradation of 2,3,3,3-tetrafluoropropionic acid by RPA1163, as evidenced by the detection of defluorination product 2-hydroxyl-3,3,3-trifluoropropionic acid using MS/MS analysis. Furthermore, Yue et al. (70) showed that RPA1163 facilitated the defluorination of difluoroacetate to glyoxylate, leading to a change in the rate-determining step for difluoroacetate degradation to C-F bond activation.

*Pseudomonas fluorescens* DSM 8341, which possesses fluoroacetate dehalogenase activity, has also been reported to degrade 6:2 FTOH and 6:2 polyfluoroalkyl phosphates (6:2 PAPs) into a range of short-chain PFASs. This finding is consistent with the involvement of fluoroacetate dehalogenase in the defluorination process of 6:2 FTOH and 6:2 PAPs. However, further investigations are needed to elucidate the molecular basis for these activities (82–84).

In a recent study conducted by Khusnutdinova et al. (74), apart from fluoroacetate, haloacid dehalogenases DAR3835, NOS0089, and POL4478 exhibited defluorination activity toward difluoroacetate, 2,2-difluoropropionic acid, and 5,5,5-trifluoropentanoic acid. The crystal structures of DAR3835 and NOS0089 resemble that of RPA1163, featuring conserved catalytic triads (Asp-His-Asp) and substrate-binding residues engaged in coordinating both the substrate fluorine and carboxylate groups. The defluorination product of difluoroacetate was identified as glyoxylate. Moreover, computational analysis of the structural characteristics of DAR3835 and NOS0089 suggested a mechanistic model wherein the enzymatic defluorination of difluoroacetate occurs through a series of two consecutive defluorination reactions. Meanwhile, the results of ligand docking analyses imply a shared catalytic mechanism for the enzymatic defluorination of both fluoroacetate and difluoroacetate (74).

The involvement of RJO0230 in the defluorination of 6:2 FTSA was implied by the observation that its expression is up-regulated in strain RHA1 grown on ethanol as a carbon source and 6:2 FTSA as a sole sulfur source (73). Additionally, its role in the defluorination of 6:2 FTOH by RJO0230 was further supported through enzyme inhibition tests and heterogeneous expression of the enzyme. Specifically, the strain RHA1, cultivated with 6:2 FTOH in the absence of CuSO_4_ (which inhibits L-2-haloacid dehalogenases), exhibited a substantially higher fluoride release compared to samples containing the inhibitor, consistent with the involvement of an L-2-haloacid dehalogenase. Subsequently, the gene (*rha1_ro00230*) encoding RJO0230 was successfully cloned and expressed in *Rhodococcus opacus* PD631 (strain PD631), which then gained defluorination activity against 6:2 FTOH as evidenced by the detection of elevated fluoride levels (73).

One gene encoding haloacid dehalogenase (PZP66635.1) from *Delftia acidovorans* was genetically modified and introduced into a pSB1C3 plasmid to enable expression in *Escherichia coli*. The transformed *E. coli* cells were subsequently cultured and exposed to 100 mg/L PFOA. During a 4-hour incubation period, the release of fluoride ions was observed in the cultures of transformed *E. coli* containing the engineered plasmid (85). Despite the absence of a statistically significant difference in fluoride ion release between the transformed *E. coli* and control samples as determined by a *t*-test, this finding has been taken as evidence of the enzyme’s potential for enzymatic defluorination.

### Reductive dehalogenases

**Fig 3:**
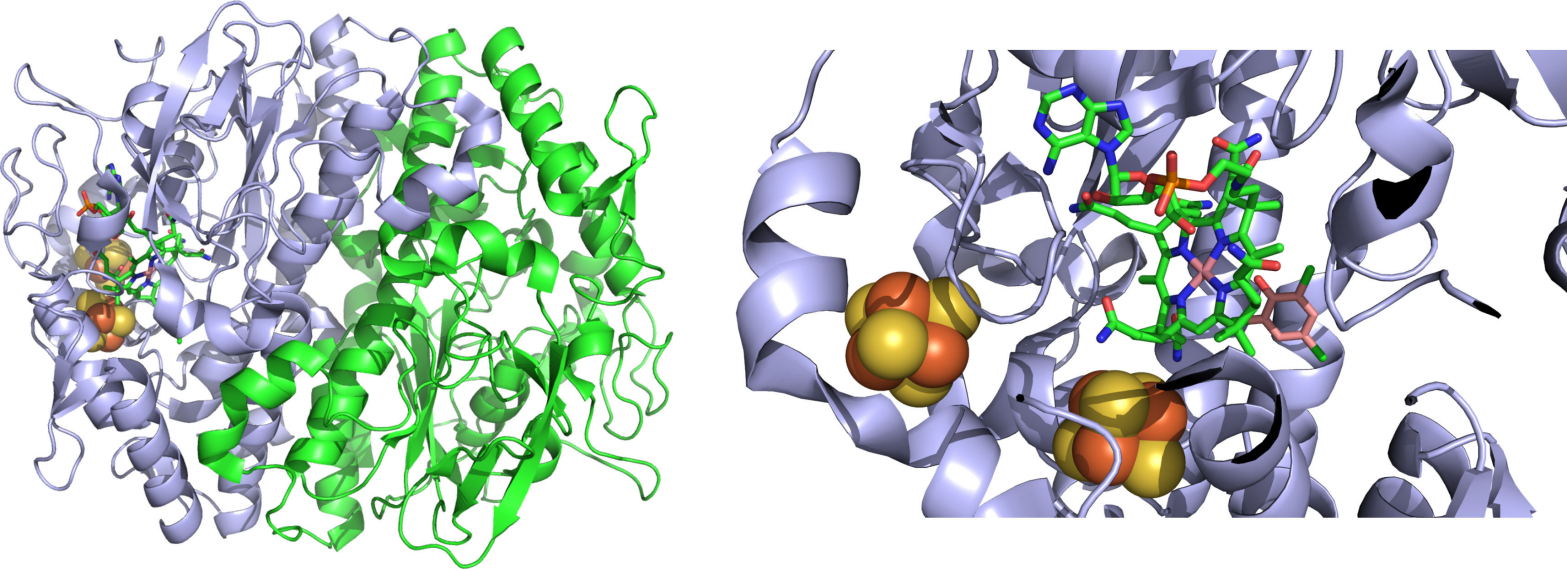
Reductive dehalogenase. Cartoon representation of the structure of the reductive dehalogenase, PCE, from *Sulfurospirillum multivorans* (PDB 5MA1) (92). The dimer is shown on the left, with the two identical chains shown in green and blue. A more detailed view of the active site is given on the right, showing the iron and sulfur of the two [4Fe-4S] clusters as red and yellow spheres, respectively. Cobalamin is shown as green sticks, and the substrate (2,4,6-tribromophenol) is shown in pink. This figure was created with BioRender.com.

Reductive dehalogenation also has the potential for defluorination of highly fluorinated organic contaminants. There are two classes of reductive dehalogenase: respiratory and catabolic. The oxygen-sensitive respiratory reductive dehalogenases use these substrates as terminal electron acceptors and are exported into the periplasm via a twin-arginine transport signal, where they form a complex with a membrane anchor protein (86–88). The catabolic reductive dehalogenases are cytoplasmic and more oxygen tolerant than their respiratory counterparts (86, 89–91). As with the hydrolytic dehalogenases, the catabolic reductive dehalogenases are thought to dehalogenate compounds that are then channeled into central carbon metabolism. Structures for both respiratory and catabolic reductive dehalogenases have been obtained, which share several similarities, including the presence of cobalamin and two [4Fe-4S] clusters in the active site (Fig. 3) (92). The reaction mechanism has not yet been fully elucidated; however, the currently favored model involves direct cobalt-halide interaction with the iron-sulfur clusters, shuttling electrons to the reaction center from a suitable electron donor.

Reductive dehalogenation of organochlorides, bromides, and iodides has been demonstrated in biochemical and microbiological studies (86). However, there is no direct evidence for reductive defluorination and relatively few reports in which this enzymatic activity has been inferred from indirect evidence; as such, the feasibility of this defluorination mechanism remains unproven and contested. One line of evidence against the feasibility of reductive defluorination of PFAS is that the redox midpoint potential of organofluorides may be too low to yield energy when used as terminal electron acceptors for biologically relevant electron donors (46). However, at least one detailed thermodynamic study suggests that reductive defluorination will yield sufficient energy to support microbial growth (93). Moreover, several studies have now demonstrated abiotic reductive defluorination of branched and unsaturated PFAS (e.g., br-PFOS and br-PFHxS) in vitamin B_12_-Ti(III) citrate catalytic system (94, 95) and vitamin B_12_-nanoscale zerovalent zinc system (96), suggesting that cobalamin-dependent respiratory reductive dehalogenases could have a role in the defluorination of organofluorides. However, it should be noted that the redox midpoint potential for Ti(III) citrate is lower than −800 mV (97), which is considerably lower than physiologically relevant electron donors, and so while this demonstrates that the corrinoid cofactor may be able to catalyze the reduction of organofluorides, there is still doubt that sufficiently low potential biological electron donors are available for this catalysis to be physiologically relevant.

Notwithstanding the unresolved question about the feasibility of biotic reductive defluorination of PFAS, several studies have presented evidence that has led to the inference of this enzymatic activity in microbial cultures. The first published study to have claimed biological reductive defluorination occurred on trifluoroacetate, with the evidence showing the generation of difluoroacetate and fluoroacetate during the biodegradation in methanogenic (anoxic) conditions (98). Similarly, Kim et al. (99) observed stepwise reductive defluorination of TFA in a long-term (90 weeks) operated anaerobic reactor. However, these results proved difficult to reproduce in methanogenic samples from different environments (100), and the microorganisms and enzymes responsible for reductive defluorination were not identified in the original studies. After over a decade, an anaerobic strain [named MFA1, formally *Cloacibacillus porcorum* strain MFA1 (101)] belonging to Synergistetes phylum was shown to defluorinate MFA but not TFA (102). MFA was stoichiometrically defluorinated to acetate with the release of fluoride. Furthermore, no fluorinated intermediates were detected in the ^19^F NMR spectrum during fluoroacetate biodegradation, yet no known fluoroacetate dehalogenases were detected in strain MFA1 (102).

Recently, Huang and Jaffé (103) reported the defluorination of PFOS and PFOA (up to 60%) by *Acidimicrobium* sp. strain A6 (belonging to the Acidimicrobiaceae family) using ammonium or hydrogen as the electron donor and iron (III) as the electron acceptor. The defluorination was evidenced by the production of fluoride ions and shorter-chain fluorinated compounds (e.g., HFBA, PFPeA, PFHxA, PFBS, and PFHpA) in the mixed culture containing *Acidimicrobium* sp. strain A6. Metagenomic analysis identified a gene encoding reductive dehalogenase subunit A (GenBank accession number: MK358462) in the genome of strain A6, which was hypothesized to be the key enzyme responsible for the defluorination of PFOA/PFOS (104), although there is no direct evidence that supports this hypothesis. Additionally, the sequence of the gene was incomplete, with a missing C-terminus of over 100 amino acids compared to other known reductive dehalogenases. However, in subsequent sequence mining using the incomplete *Acidimicrobium* sp. strain A6 gene (referred to as A6RdhA), Guo et al. (105) found a full-length gene encoding a protein named T7RdhA from the *Acidimicrobium* strain TMED77 in a metagenomic assembly of marine microorganisms that shares 97.67% sequence identity with the partial A6RdhA protein. T7RdhA was identified as a corrinoid iron-sulfur protein that employs a norpseudo-B_12_ cofactor and two Fe_4_S_4_ iron-sulfur clusters for catalytic activity (106), through AlphaFold2 modeling and experiments. Moreover, results from docking and molecular dynamics simulations implied that T7RdhA could potentially use PFOA as a substrate (105). As with A6RdhA, there is no empirical evidence that supports a role for this protein in PFAS defluorination.

In 2020, Yu et al. (107) inferred the reductive defluorination of two C_6_ unsaturated and branched fluorinated compounds by a *Dehalococcoides*-containing trichloroethene-dechlorinating consortium (KB1). Specifically, in addition to lactate as the electron donor and the fluorinated compounds as the sole electron acceptor, vitamin B_12_ (100 µg/L) was supplemented to KB1 as the essential cofactor for dechlorinating members. The detection of released fluoride ions and corresponding products was attributed to reductive defluorination. Moreover, Yu et al. (108) demonstrated the generation of fluoride in the presence of two (C_5_ and C_8_) unsaturated fluorinated compounds by KB1 under the same culture conditions. Regarding reductive defluorinases, no gene transcription of reductive dehalogenases in KB-1 was observed during the defluorination of fluorinated compounds (107). To date, the identities of the enzymes responsible for defluorination are still unknown.

### Cytochromes P450

Cytochromes P450 (P450s or CYPs) are a widely distributed superfamily of enzymes found in all three domains of life (Archaea, Eubacteria, and Eukaryotes). They are heme-thiolate proteins characterized by the linkage of the heme prosthetic group to the apoprotein through a conserved axial cysteine residue. In the presence of molecular oxygen and the reduced cofactor NAD(P)H, the majority of P450s exhibit catalytic activity in monooxygenation reactions. The classical catalytic cycle of P450 involves a series of sequential steps, encompassing the reductive activation of molecular oxygen through sequential single-electron transfers to the iron-active site, its heterolytic cleavage, and the subsequent formation of the hydroxylated product (109–111).

**Fig 4:**
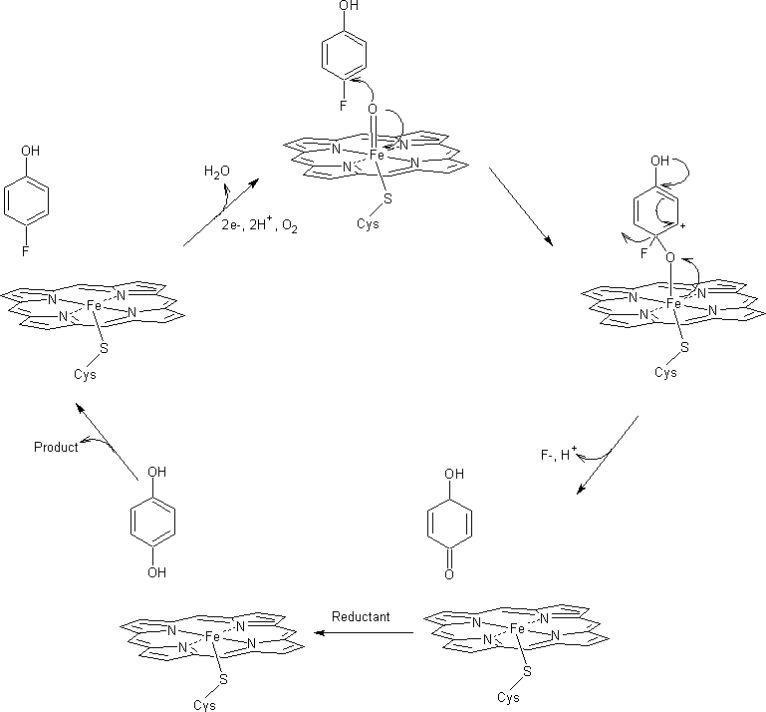
Overview of the cytochrome P450-mediated defluorination of 4-fluorophenol. 4-Fluorophenol binds to complex I, which displaces fluoride via attack of the oxygen of complex I on the fluorinated carbon to form a semiquinone, which is then reduced to form 4-hydroxyphenol.

Organic pollutants are among the wide range of organic compounds known to be substrates for naturally occurring and engineered P450s [reviewed by Lin et al. (112)]. In particular, some P450s can perform oxidative and reductive dehalogenations. For instance, P450 from livers of Wistar rats catalyzed the oxidative defluorination at the para position of pentafluorophenol or 4-fluoroaniline, releasing fluoride ion and generating tetrafluorobenzoquinone or 4-hydroxyaniline (113–115). P450 2E1 from human livers exhibited oxidative defluorination activity on fluorinated inhalation anesthetics (e.g., sevoflurane, isoflurane, and methoxyflurane), resulting in the generation of toxic metabolic intermediates (116, 117). Furthermore, P450_BM3_-F87G, a mutant form of P450_BM3_ from *Bacillus megaterium*, catalyzed the oxidative defluorination of 4-fluorophenol to benzoquinone that was further reduced to hydroquinone through the NADPH P450-reductase (118) (Fig. 4). Dehalogenation by P450s relies on the availability of reducing equivalents, the reduction of the heme iron, and the dynamic interplay of the active site, which ensures the appropriate positioning of the substrate in close proximity to the reduced heme (119). Li and Wackett (120) conducted *in vitro* assays using P450_CAM_ from *Pseudomonas putida* in the presence of titanium (III) citrate as electron donor and organohalides as electron acceptors. Specifically, 1,1,1-trichlorotrifluoroethane (Cl_3_CCF_3_) was reductively dehalogenated to a mixture comprising 1,1-dichlorodifluoroethylene (Cl_2_C═CF_2_) and 1,1-dichloro-2,2,2-trifluoroethane (HCl_2_C-CF_3_) in nearly equal proportions. In contrast, the reductive dehalogenation product of trichlorofluoromethane (FCCl_3_) was identified as carbon monoxide (CO). This reaction was proposed to occur through intermediates of dichlorofluoromethyl (FCCl_2_) and chlorofluorocarbene (FCCl) radicals, with the chlorofluorocarbene radical subsequently reduced to CO by water (120).

In terms of PFAS defluorination, P450 from strain RHA1 was implicated in the defluorination of 6:2 FTOH (73); it exhibited a significantly higher rate of release of fluoride when cultivated with 6:2 FTOH in the absence of P450 inhibitors (1-aminobenzotriazole and allylthiourea) than when those inhibitors were present. Subsequently, the gene responsible for encoding the responsible P450 was successfully cloned and expressed in strain PD631, which then gained defluorination activity on 6:2 FTOH. Moreover, the gene encoding this P450 also exhibited statistically significant expression during the defluorination process of 6:2 FTSA when strain RHA1 was supplied with ethanol and 6:2 FTSA as carbon and sulfur sources (73).

P450s from fungi (such as *Cunninghamella elegans* and *Phanerochaete chrysosporium*) have also been reported to participate in the biotransformation of 6:2 FTOH (121–123). Khan and Murphy (121) observed that *C. elegans* failed to transform 6:2 FTOH in the presence of the P450 inhibitor (1-aminobenzotriazole and allylthiourea), supporting a role for P450s in this biotransformation process. Moreover, they found the inhibition of P450 activity by 5:3 FTCA, the primary metabolite accumulated during the biotransformation of 6:2 FTOH (121). Merino et al. (122) quantified NADPH-P450 reductase (CPR) activity to indirectly assess the whole P450 catalytic system during the biotransformation of 6:2 FTOH by *P. chrysosporium*. They noted that CPR activity significantly increased in the presence of 6:2 FTOH compared to its absence, suggesting the engagement of P450s in the biotransformation of 6:2 FTOH. However, the specific function of P450s in fungal 6:2 FTOH biotransformation remains unclear. Khan and Murphy (123) demonstrated that heterologously expressed CYP5208A3 and CYP reductase B (CYP5208A3/CPR_B) from *C. elegans* exhibited the capability to convert 6:2 FTOH to 6:2 FTCA in whole-cell assay using yeast *Pichia pastoris* X-33.

### Alkane and butane monooxygenases

Several monooxygenases can catalyze the oxidation of alkanes, with soluble butane monooxygenase and alkane monooxygenase being among the enzymes that have been characterized (124, 125). One extensively investigated example of a soluble butane monooxygenase is the three-component diiron monooxygenase complex from *Pseudomonas butanovora*. This complex comprises a hydroxylase in an α_2_β_2_γ_2_ configuration, a reductase that transfers electrons from NADH to the active site in the hydroxylase α-subunit, and a regulatory protein (126). Butane monooxygenase has been found to confer cometabolic dechlorination activity in the aerobic cultures grown on alkanes (127–129), in addition to its physiological function to oxidize alkanes (C_2_–C_9_) and alcohols (C_2_–C_4_) (130). As an integral membrane-bound diiron *ω*-hydroxylase, alkane monooxygenase from *Pseudomonas putida* Gpo1 (formerly known as *Pseudomonas oleovorans*) can selectively introduce O_2_ into the unreactive terminal methyl group of C_5_–C_12_ linear alkanes, generating primary alcohols (131, 132).

Based on the structural similarity shared between *n*-alkanes and FTOHs, it was postulated that alkane-degrading enzymes, particularly butane and alkane monooxygenases, may possess the ability to degrade FTOHs as well (133). Two strains that possess butane and alkane monooxygenases have been shown to catalyze the defluorination of FTOHs (i.e., 4:2, 6:2, and 8:2 FTOHs) and 6:2 polyfluoroalkyl phosphates (6:2 PAPs) by eliminating multiple –CF_2_– groups, ultimately resulting in the formation of shorter-chain PFCAs (82–84, 133). While butane monooxygenase has been suggested to play a role in the defluorination of FTOHs, further investigations are essential to clarify its precise involvement (84). In contrast, there is evidence to support the participation of alkane monooxygenase in the defluorination of both FTOHs and 6:2 PAPs (82, 83). High gene copy numbers of *alkB*, the gene encoding alkane monooxygenase, have been positively correlated with high levels of fluoride released from the biodegradation of both FTOHs and 6:2 PAPs (82, 83).

Similarly, Yang et al. (73) confirmed the involvement of alkane monooxygenase in strain RHA1 in the defluorination of 6:2 FTOH through inhibition tests and heterogeneous expression. Specifically, the 6:2 FTOH-grown strain RHA1 devoid of acetylene and allylthiourea (inhibitors of alkane monooxygenase) exhibited significantly greater quantities of fluoride released compared to those samples containing enzyme inhibitors. The gene responsible for encoding alkane monooxygenase was cloned and expressed in strain PD631. This genetically engineered strain PD631 exhibited defluorination activity on 6:2 FTOH, as evidenced by the detection of high levels of fluoride released. Furthermore, this gene encoding alkane monooxygenase also demonstrated a statistically significant expression in the defluorination of 6:2 FTSA when strain RHA1 was fed with ethanol as a carbon source and 6:2 FTSA as the sole sulfur source (73).

### Peroxidases

Peroxidases, heme-containing enzymes, are ubiquitously distributed across fungi, bacteria, plants, and animals. By employing hydrogen peroxide (H_2_O_2_) or organic hydroperoxides as co-substrates, these enzymes facilitate oxidative reactions involving numerous inorganic and organic substrates (134, 135). Many peroxidases are excreted into the environment to catalyze the decomposition of complex organic polymers, such as cellulose and lignin, by saprotrophic organisms (136).

Although these enzymes exhibit varying specificity toward oxidizable substrates, they follow a shared catalytic cycle. Upon interaction with an H_2_O_2_ molecule, enzymatic reactions proceed via a sequence of three successive redox steps. First, the enzyme undergoes oxidation, forming a cation radical (compound I) and concurrently reducing H_2_O_2_ to water. Subsequently, compound I is reduced, facilitating the oxidation of an organic substrate and the formation of compound II and an organic radical. Finally, compound II undergoes reduction to return to its resting state, accompanied by the oxidation of a second substrate molecule and generation of another organic radical (135). The generation of free radicals during this process is accountable for the degradation of pollutants into smaller biodegradable products with minimal toxicity (134). Consequently, peroxidases play a crucial role in catalyzing the biodegradation of diverse contaminants, including pesticides, phenolic pollutants, textile dyes, polycyclic aromatic hydrocarbons, and polychlorinated biphenyls [reviewed by Bilal et al. (137) and Sellami et al. (138)]. Among them, extracellular peroxidases including horseradish peroxidase (HRP), lignin peroxidase (LiP), and manganese peroxidase (MnP) have been documented to engage in the biodegradation and biotransformation of PFAS.

#### HRP

HRP, extracted and obtained from horseradish roots, can catalyze the oxidation of phenolic acids, aromatic phenols, and non-aromatic amines using H_2_O_2_ (139). Interactions between HRP and phenolic substrates facilitate the generation of highly reactive radical intermediates, enabling catalysis of secondary reactions with diverse organic contaminants (140). Hence, in addition to its application in the degradation of phenol-containing wastewater, HRP has also been extensively employed for the transformation/degradation of dyes, pesticides, pharmaceuticals, and various hazardous contaminants (137, 138).

Colosi et al. (140) first reported the effectiveness of HRP-mediated degradation of PFOA. In reactions using HRP, H_2_O_2_, and 4-methoxyphenol (a phenolic co-substrate), approximately 68% removal of PFOA (initial concentration of 850 µM) was observed. Accompanied by PFOA degradation, fluoride ions (less than 1%) and various short-chain fluorinated compounds were identified via ion chromatography and gas chromatography-mass spectrometry, respectively.

#### LiP and MnP

LiP and MnP are capable of catalyzing lignin degradation in the presence of H_2_O_2_. In addition to following a similar catalytic cycle (e.g., HRP or LiP), MnP uses Mn^2+^ ions as electron donors, wherein these ions are oxidized to Mn^3+^ ions using H_2_O_2_. This oxidative transformation is a crucial step contributing to lignin degradation and activation of the ligninolytic system (134, 141). Both LiP and MnP can oxidize a wide range of environmental pollutants such as phenolic and non-phenolic compounds, dyes, and xenobiotics (134, 139, 142).

The genes encoding LiP (*lipD*) and MnP (*mnp1*) demonstrated differential expression in comparison to the PFAS-free control when *P. chrysosporium* was cultured under ligninolytic conditions in a modified Kirk medium containing 1% glucose (122). Specifically, both genes exhibited gradual accumulation of transcripts over time, reaching their peak expression levels on day 28 when *P. chrysosporium* was cultivated with 5:3 FTCA as the parent compound. Correspondingly, the LiP activity exhibited a progressive increase over time, whereas the MnP activity remained stable between days 7 and 14, followed by an upsurge on day 28. These observations suggest that both enzymes may have contributed to the biotransformation of 5:3 FTCA or were positively influenced by 5:3 FTCA and its metabolites. The underlying mechanisms warrant further investigation in future studies (122).

Exploration of the defluorination mechanisms of PFOA and PFOS driven by peroxidases is currently at an early stage. These mechanisms were inferred based on the identified products and theoretical calculations (140). For HRP-driven PFOA degradation, the initial assumption was that PFOA underwent Kolbe decarboxylation, followed by the stepwise conversion of -CF_2_ units to CO_2_ and fluoride ions. This speculation was based on the detected products and fluoride ions. However, there was a substantial imbalance in the released fluoride concentrations between the actual measured value (2 mg/L) and theoretical value (165 mg/L) (140), suggesting that further work is needed to understand this reaction at a mechanistic level.

### Laccases

Laccases, primarily obtained from bacteria, fungi, plants, and insects, constitute a subgroup within the enzyme family of multi-copper oxidases (143). Like the peroxidases, extracellular laccases are used by saprophytes in the decomposition of complex biological polymers, such as polysaccharides and lignin (136). Within their active sites, these enzymes harbor four copper atoms, categorized into three types: single “blue” copper (type 1, T1), single “non-blue” copper (type 2, T2), and a copper-copper pair (type 3, T3) (143, 144). Laccases exhibit the capability to oxidize a wide range of phenolic and non-phenolic compounds and employ O_2_ as an electron acceptor (four-electron reduction of oxygen), yielding water as a by-product (143). This renders them extensively applicable in the oxidative removal of various pollutants, such as herbicides, synthetic dyes, and pharmaceuticals (134, 143, 144).

Laccases catalyze the oxidation of various substrates via a single-electron transfer, involving a chemical mediator. Specifically, they oxidize suitable redox mediators through a single-electron transfer mediated by the copper site, forming a free radical cation. Subsequently, an internal electron is transferred from the reduced T1 to the T2 and T3 copper sites, where the bound O_2_ molecule undergoes activation and reduction to water in the T2/T3 trinuclear domain (145, 146). This radical cation formed by mediator oxidation diffuses from the enzyme-active site and non-specifically oxidizes reduced compounds in the local environment. Examples of such mediators include 2,2′-azino-bis(3-ethylbenzothiazoline-6-sulfonic acid) and 1-hydroxybenzotriazole (HBT) (144, 145).

Luo and co-authors explored the degradation of PFOA and PFOS through the enzyme-catalyzed oxidative humification reactions (ECOHRs) using laccase (147–150). Laccase (sourced from *Pleurotus ostreatus*) effectively degraded approximately 50% (0.5 µM) of PFOA over 157 days, with periodical addition of HBT. Fluoride ions were detected, and the concentration revealed 28% defluorination of PFOA. Rather than short-chain perfluorocarboxylic acids, partially fluorinated shorter-chain alcohols and aldehydes were identified as degradation products via high-resolution mass spectrometry (147). The metal ion regulation of PFOA and PFOS degradation in the laccase-HBT system was investigated (148, 149). Notably, Cu^2+^ and Fe^3+^ can complex with PFOA, thereby enhancing PFOA degradation and increasing the HBT radical efficacy. In contrast, little PFOA degradation occurred in solutions containing Mg^2+^ or Mn^2+^ (148). Metal ions, Mg^2+^ and Cu^2+^, were also tested with PFOS degradation in the laccase-HBT system, resulting in 59% (0.59 µM) degradation in the Cu^2+^ solution and 35% (0.35 µM) in the Mg^2+^ solution over 162 days (149). The products of PFOS degradation were identified as partially fluorinated compounds, similar to those reported in previous studies of PFOA degradation during ECOHRs by Luo et al. (147, 148). The presence of metals capable of complexing with the selected PFAS was postulated to reduce the distance between laccase and the negatively charged PFOS and PFOA, thereby increasing the likelihood of the HBT radical reacting with them (148, 149). Instead of HBT, soybean meal was validated as an alternative organic mediator in laccase-induced ECOHR for PFOA degradation in soil (150). Moreover, purified laccase (*Pleurotus ostreatus*, purchased from Sigma-Aldrich) and crude laccase (concentrated from the fungus *Pycnoporus* sp. SYBC-L3) exhibited similar efficiency in PFOA degradation (150).

As is the case for peroxidase-mediated defluorination, in laccase-mediated PFOA and PFOS degradation, the generation of partially fluorinated short-chain compounds was attributed to degradation via a combination of free-radical decarboxylation, coupling, and rearrangement processes (147, 149, 150), although more evidence is needed to understand this mechanism.

### Desulfonases that may participate in fluorotelomer biodegradation

**Fig 5:**
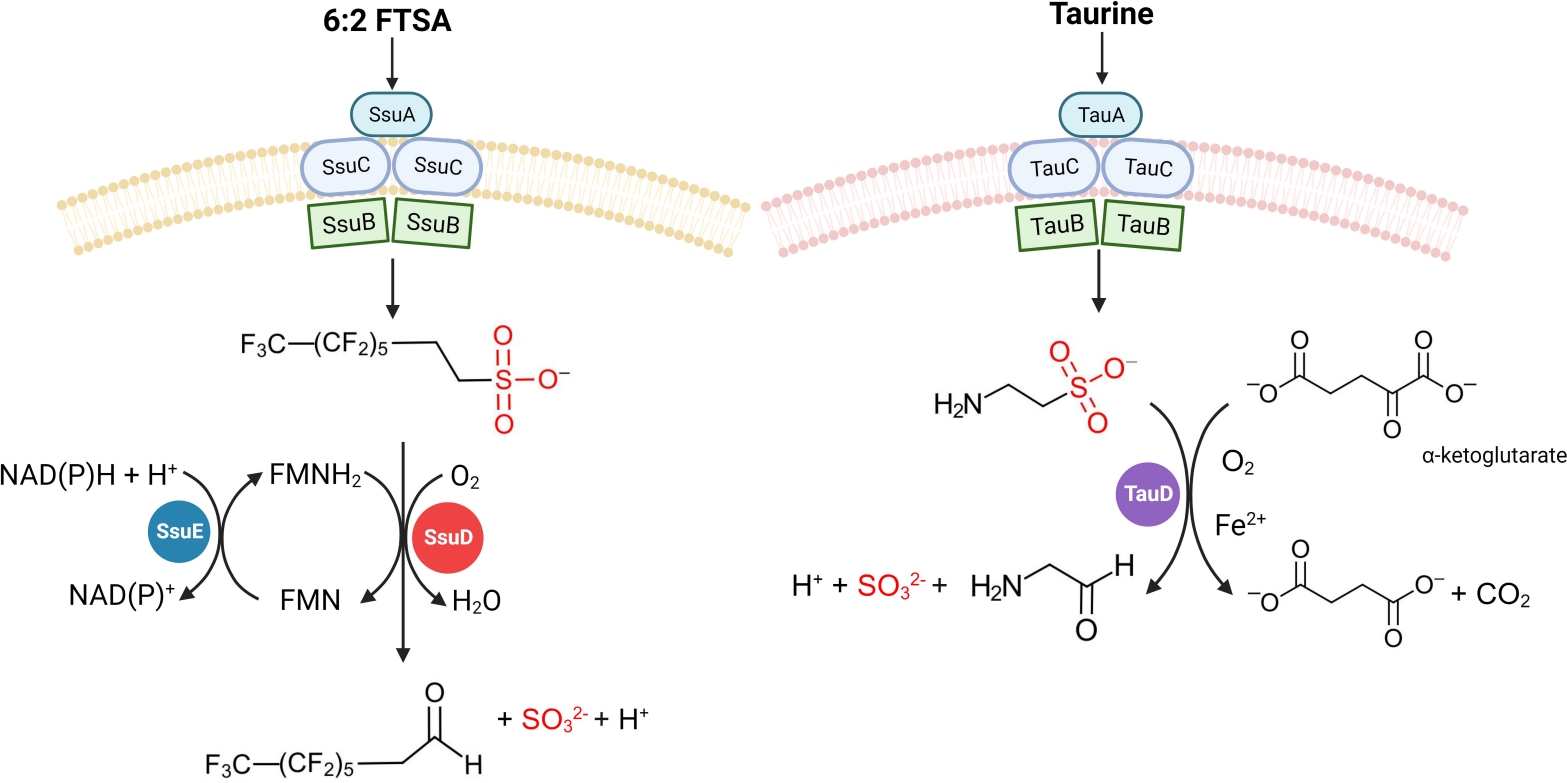
Schematic regarding uptake and desulfonation of alkanesulfonates and taurine by (**A**) TauABCD and (**B**) SsuEADCB systems, based on data from van der Ploeg et al. (153). This figure was created with BioRender.com.

Sulfur serves as an essential component of amino acids and enzyme cofactors in all. Numerous bacterial organisms acquire sulfur via the assimilation pathway of inorganic sulfate or amino acid sulfur, which ultimately yields sulfide, subsequently utilized in the synthesis of sulfur-containing organic molecules (151–153). However, inorganic sulfur sources are not available for bacteria to grow in all environments. Consequently, bacteria have evolved an ability to acquire sulfur from organosulfonates such as sulfonates and sulfonate esters, both aerobically and anaerobically [reviewed by Kertesz (151)]. Under sulfur-limiting conditions, certain bacteria express two operons, *tau* and *ssu*, to utilize organosulfonates as sulfur sources for growth. Both operons encode an ABC-type transport system for the uptake of organosulfonates (*tauABC* and *ssuABC*) and an oxygenase system for their desulfonation (*tauD* and *ssuDE*) (Fig. 5). Consistent with their metabolic function, the Tau and Ssu proteins demonstrate a lower-than-average content of sulfur-containing amino acid residues (151, 153–155). In addition, the TauABC and SsuABC transport systems are not interchangeable due to no shared membrane component or ATPase between the periplasmic binding proteins of these two systems (153, 156).

TauD is an Fe (II) and α-ketoglutarate-dependent dioxygenase, with activity with a range of sulfonates (e.g., butanesulfonate and pentanesulfonate), but a preference for taurine (153, 157). TauD catalyzes the oxygen-dependent desulfonation of taurine into sulfite and aminoacetaldehyde, with the unstable intermediate 1-hydroxyaminoethanesulfonate presumed as an intermediate (157) (Fig. 5).

SsuD is an alkanesulfonate monooxygenase belonging to a two-component system that catalyzes the desulfonation of organosulfonates to sulfite and the corresponding aldehyde (153, 154, 158) (Fig. 5). Its activity is dependent on reduced flavin mononucleotide (FMNH_2_) that is provided by SsuE (an NAD(P)H-dependent FMN reductase) (158), where the reduced flavin functions as a co-substrate, instead of a bound prosthetic group (151, 154). SsuD has a broad substrate range, including linear alkanesulfonates (from methanesulfonate to tetradecanesulfonate) and several substituted sulfonated compounds, but not taurine (158, 159). Recently, it was suggested that 6:2 FTSA is also included into the substrate range of SsuD, albeit the evidence supporting this claim is incomplete. Strain RHA1, a pure strain isolated from γ-hexachlorocyclohexane-contaminated soil, is capable of desulfonating and defluorinating 6:2 FTSA, where 6:2 FTSA is used as a sole sulfur source to support growth (73). Additionally, the gene *ssuD* exhibited a significantly higher expression level in strain RHA1 when supplemented with ethanol and 6:2 FTSA as carbon and sulfur sources, respectively, in comparison with the control group that adopted an identical carbon source with sulfate as the sole sulfur source. Furthermore, genes (*ssuD* and *ssuE*) were expressed in strain PD631 (*ssuD*) and *E. coli* BL21 (DE3) (*ssuE*), respectively. Sulfite (60–100 µM) was detected in the reaction mixture containing crude enzymes of SsuD/SsuE and 6:2 FTSA (500 µM) with 1 hour incubation at 30℃ (73). However, the aldehyde product was not observed in these reactions, which is necessary to confirm the desulfonation of 6:2 FTSA by SsuD/SsuE in strain RHA1.

Nitrilotriacetate monooxygenase (NTA-Mo) has also been suggested to desulfonate 6:2 FTSA and fluorotelomer sulfonamidoalkyl betaine (6:2 FTAB) (160–163). NTA-Mo belongs to the family of two-component monooxygenases as SsuD/SsuE, functioning as an oxidoreductase for initiating the oxidation of NTA under aerobic conditions (164, 165). This enzyme consists of two components, NtaA and NtaB (165). The former exhibits monooxygenase activity, catalyzing the oxidative conversion of NTA to iminodiacetate (IDA) and glyoxylate in the presence of FMNH_2_ and O_2_. Meanwhile, the latter, a flavin reductase, utilizes NADH to reduce FMN to FMNH_2_, a requisite cofactor in the oxidization step by NtaA (165).

*Gordonia* sp. strain NB4-1Y, able to cleave C-S bonds, showed activities in desulfonation and partial defluorination of 6:2 FTSA and 6:2 FTAB (160, 161, 163). Transcriptomics analysis revealed that in addition to one gene encoding alkanesulfonate monooxygenase (RS02630), two nitrilotriacetate monooxygenase genes (RS14730 and RS14155) were highly expressed in the presence of 6:2 FTSA and 6:2 FTAB compared with MgSO_4_ as sources of added sulfur for growth (161). Previously, Van Hamme et al. (160) found that two nitrilotriacetate monooxygenases (component A) (*ntaA*) were differentially expressed in the presence of 6:2 FTSA through two-dimensional differential in-gel electrophoresis (2D DIGE) experiments on strain NB4-1Y growing in the 6:2 FTSA (100 µM) or MgSO_4_ (100 µM) as the sole sulfur source, respectively. Subsequently, they proposed that these two NtaAs functioned as desulfonases. In fact, through the genomic re-annotation and transcriptomics analysis, these two NtaAs, highly expressed in the cultures of strain NB4-1Y amended with 6:2 FTAB and 6:2 FTSA, were re-annotated as two FMN-dependent monooxygenases (RS09755 and RS 09775) (161). Consistent with the hypothesis that these genes encode desulfinases, the encoded proteins have significantly lower percentages of sulfur-containing amino acids that would be expected by chance (155, 160, 161).

The genome of *Dietzia aurantiaca* J3 (strain J3), isolated from landfill leachate exposed to some PFAS (e.g., 6:2 FTSA, PFOS, PFOA, and PFHxS), contains an operon related to uptake and assimilation of organosulfonates. Proteomics, where strain J3 grew amended with 6:2 FTSA and MgSO_4_ as sulfur sources, respectively, revealed that putative enzymes capable of transporting and desulfonating 6:2 FTSA were significantly upregulated (162). For the putative desulfonase annotated as *ssuD* in strain J3 (MCD2262844.1) (162), it actually has over 99% identity with luciferase-like monooxygenase class flavin-dependent oxidoreductase (WP_269074981.1) and over 89% identity with alkanesulfonate monooxygenase (PAY22235.1) in *Dietzia* sp. The *ntaB* annotated based on NCBI Prokaryotic Genome Annotation Pipeline (MCD2262842.1) shares 100% identity with flavin reductase family protein (WP_230929925.1) in *Dietzia aurantiaca*, which provides reduced flavin for desulfonation.

Notably, the assertion that strains NB4-1Y and J3 may use desulfonases in the catabolism of PFAS is supported largely by the correlation between changes in their transcriptomes and the presence of PFAS in their growth media. Moreover, neither strain is able to grow with PFOS or PFHxS as sole sulfur sources (160, 162). Further investigation is essential to provide direct evidence for PFAS desulfonation and the desulfonases identified.

## OTHER MOLECULAR PROCESSES NEEDED TO SUPPORT MICROBIAL PFAS DEFLUORINATION

### Resistance to intracellular fluoride

A major concern for the microbial remediation of fluorinated compounds is the production of intracellular fluoride. The anion is highly toxic to microbes, impacting the function of a variety of metabolic processes, such as enolase activity (166), cation translocation (167), and polysaccharide synthesis (168). It also behaves as a weak acid itself, accumulating in the cytoplasm without active uptake when the extracellular pH is lower than the intracellular pH, due to the high pK_a_ and membrane permeability of hydrogen fluoride (HF) (169).

Fluoride is abundant in the Earth’s crust and present in most, if not all, natural environments. To counter the toxic effects of fluoride, bacteria have evolved inducible, fluoride-selective export channels. The first evidence of a molecular mechanism for bacterial resistance to fluoride was the discovery of a widespread family of fluoride-responsive riboswitches, found upstream of genes predicted to encode halide-specific membrane channels (170–172). When coupled to a reporter system, the fluoride riboswitch has also been shown to have utility as a sensitive and biocompatible fluoride sensor that can be used in living bacteria or in wholly *in vitro* systems (170, 173).

**Fig 6:**
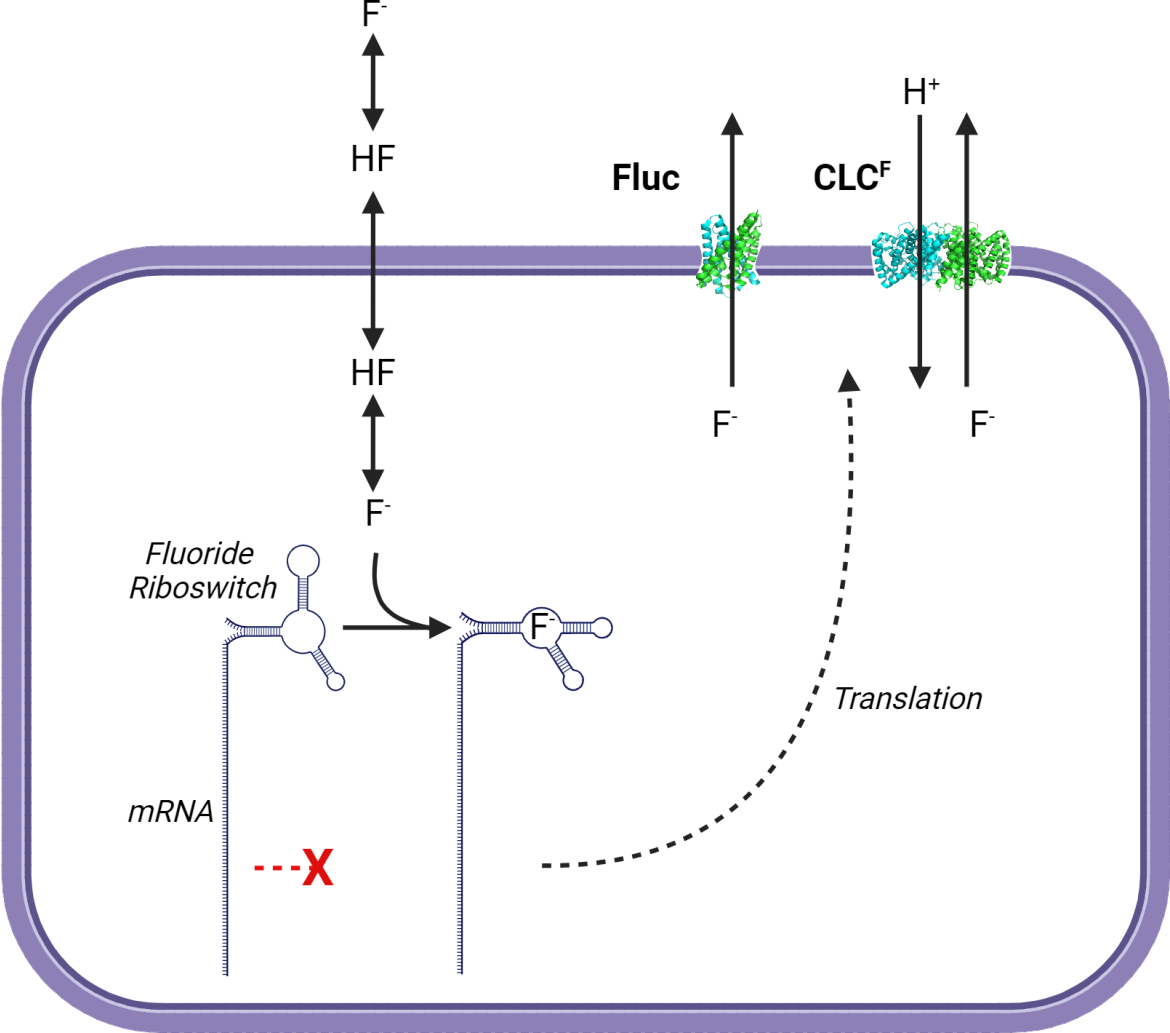
Bacterial response to fluoride toxicity. Hydrogen fluoride and fluoride (F^−^) are present in pH-dependent equilibrium, and only HF can cross biological membranes without the aid of efflux channels. At low environmental pH, this drives the accumulation of intracellular F^−^. F^−^ -responsive riboswitches bind F^−^, allowing the translation of F^−^-specific efflux proteins, including Fluc F^−^ channel (PDB: 6B2A) (177) and the H^+^/F^−^ CLC^F^ antiporter (PDB: 36D0J) (178). This figure was created with BioRender.com.

There are two known classes of F^−^ channels, which appear to have evolved independently of each other: fluoride-specific members of the CLC family and the Fluc family of proteins. The broader CLC family members are chloride transporters that are found in every domain of life. A subclass of bacterial CLC proteins has been described that lack the conserved chloride-binding motif and that were expressed under the control of a fluoride-inducible riboswitch. These CLC^F^ proteins have been demonstrated to be F^−^/H^+^ antiporters with high specificity for F^−^ compared with other anions (174), unlike chloride-specific CLCs that tend to have more promiscuous activities. Moreover, expression of CLC^F^ has been demonstrated to confer resistance to fluoride toxicity, supporting the hypothesis that F^−^ efflux is its physiological function (172). The Fluc proteins are small, homodimeric permeases (175), with extremely high specificity for the fluoride anion (10,000-fold versus chloride) (176). This ultrahigh specificity has been suggested to allow the permease to remain “open” at all times without leading to leakage of ions that would otherwise result in the collapse of the membrane potential (176) (Fig. 6).

### PFAS uptake

The majority of enzymes discussed in this review are either intracellular or membrane bound. For these enzymes to interact with PFAS as a substrate, PFAS needs to cross cellular membranes. However, there is a significant knowledge gap regarding the mechanisms underlying cellular PFAS uptake, particularly in microbes. In recent years, numerous studies have emerged and subsequently reviewed the processes of PFAS uptake and transportation in plants (18, 179–186). In general, PFAS uptake by plants occurs through root uptake and foliar uptake, with the former widely regarded as the primary pathway (181, 183, 184) and the latter potentially for semi-volatile PFAA precursors and their degradation products (such as FTOHs) (180, 183).

The pathways associated with PFAS uptake by roots and their subsequent transportation within plants are intricate and species dependent (18, 179, 184, 185), influenced by various factors, such as PFAS properties (180, 185, 186), and environmental factors (such as pH, temperature, and soil organic carbon) [reviewed by Adu et al. (186), Mei et al. (184), and Ghisi et al. (18)]. Moreover, proteins in plant roots have been identified in various studies as factors influencing the processes of PFAS uptake and accumulation (179, 185, 186). For example, Wen et al. (187) explored the plant uptake mechanism of PFOS and PFOA in maize, finding that PFOA uptake by maize roots is an active (energy-dependent) process, potentially involving anion channels, whereas PFOS uptake is a passive (carrier-mediated) process, potentially occurring through aquaporins and anion channels in root cell membranes. Wang et al. (188) investigated the uptake process of PFOA and PFOS in the wetland plant *Alisma orientale*, revealing active absorption facilitated by water and anion channels in the roots.

In addition, short-chain PFAS tend to accumulate in plant leaves because of their small molecular size and relatively high water solubility. This feature facilitates their ease of passage through root cell walls, leading to higher translocation and bioaccumulation potentials (180, 186). In contrast, long-chain PFAS are more likely to accumulate in roots, exhibiting a higher adsorption affinity due to their hydrophobic nature (185, 186). Differences in PFAS uptake and translocation are also affected by the functional head groups, leading to observable distinctions in the uptake and translocation patterns of PFCAs and PFSAs (189, 190).

## OUTLOOK

Excepting the evolutionary response to highly toxic compounds (i.e., fluoroacetate), the low abundance and diversity of naturally occurring fluorinated compounds in the environment have led to the lack of selective pressures that would otherwise have supported the evolution of biological defluorination. While biological defluorination has some unique challenges, rigorous investigation into the biochemical routes to defluorination of organofluoride compounds suggests that there is no specific physiochemical impediment to the natural evolution of such biological systems.

PFAS were first deployed over 70 years ago in the 1950s. Pesticides, such as triazine herbicides and organophosphate insecticides, were introduced in the same 1950s and 1930s, respectively, and microbial degradation of these compounds has been observed and characterized in molecular detail since before the start of this millennium (33, 191). None of these compounds are toxic to bacteria but instead provide access to otherwise limited nutrients, providing a selective pressure by virtue of the growth advantage their catabolism provides (192).

PFAS could potentially provide a carbon source after defluorination, but to access any carbon atom in the molecule requires the removal of up to three fluorides. It is very likely that multiple enzymes with promiscuous defluorination activity would be required within a bacterium or bacterial community to achieve complete defluorination of a PFAS molecule and provide a starting point for the evolution and assembly of dedicated PFAS catabolic pathways. While this may be technically tractable, the growth advantage for doing so may not provide a large enough competitive advantage to drive this evolutionary outcome.

It has also been suggested that PFAS could be used as a terminal electron acceptor in anaerobic respiration, providing a significant growth advantage under anaerobic conditions. Some early reports provide circumstantial evidence that this energy metabolism pathway may be present in *Acidimicrobium* and other bacterial strains (103, 107, 108), while some researchers suggest that existing electron transport chains may be unsuitable for the delivery of electrons to fluorinated compounds due to their extremely low redox mid-point potentials (as low as −2,700 mV) (47). There is a clear need to provide direct evidence for respiratory defluorination of PFAS to establish whether or not these pathways are possible.

Regardless of the molecular mechanisms proposed, many of the reports of PFAS biodegradation to date rely on indirect or incomplete evidence to support their claims. The formation of fluoride or reduction in concentration of substrate is an insufficient line of evidence to support claims of PFAS biodegradation. Instead, all the reaction products (not just fluoride) need to be identified. Where practical, time courses that quantify the formation of these products and reduction in substrate concentration should also be presented to demonstrate mass balance and eliminate the possibility of adsorption/absorption of the substrate.

Similarly, for claims involving the identification of gene/enzyme systems and molecular mechanisms of defluorination, direct evidence is needed. The presence of homologs of genes that encode proteins that are mechanistically plausible candidate PFAS degraders is not sufficient. Genetic knockouts, complementation studies, functional heterologous expression, and *in vitro* studies all provide direct evidence for the involvement of specific gene/enzyme systems, and ideally, multiple lines of evidence should be provided.

Fluoride toxicity and resistance mechanisms are well understood and will be necessary for bioremediation using live organisms. However, there is a substantial knowledge gap concerning PFAS uptake in microorganisms. While the use of extracellular enzymes may mitigate the need for PFAS uptake, this strategy precludes the use of enzymes that require intracellular cofactors (e.g., NAD(P)H) to function. A detailed mechanistic understanding of PFAS transport across biological membranes will greatly improve our ability to engineer solutions for environmental PFAS contamination.

Regardless of the reason, catabolism of PFAS appears not to be a widespread capability in microbial communities. However, as the biochemical potential for PFAS defluorination is clear, synthetic biology and enzyme engineering may afford non-natural approaches to approaches to developing PFAS bioremediants. Rapid advances in artificial intelligence and machine learning are already enabling substantial advances in biological design that were unimaginable 5 years ago (193–196), and the application of these techniques to the PFAS problem has already started to appear in the scientific literature (197–199). As discussed in this review, the progenitor “biochemical tool kit” for the biodegradation of PFAS and other highly fluorinated anthropogenic compounds already exists; whether a synthetic approach or evolutionary pressures are first to produce a biological solution to the issue of PFAS in the environment is still an open question.
